# Circular RNA: A promising new star for the diagnosis and treatment of colorectal cancer

**DOI:** 10.1002/cam4.4398

**Published:** 2021-11-18

**Authors:** Shunhao Zhang, Jing Sun, Minqi Gu, Guihua Wang, Xudong Wang

**Affiliations:** ^1^ Graduate School of Nantong University Nantong China; ^2^ Department of Laboratory Medicine Affiliated Hospital of Nantong University Nantong China

**Keywords:** biomarker, cancer stem cell, circular RNA, colorectal cancer, recurrence and metastasis, therapeutic target

## Abstract

**Background:**

Colorectal cancer (CRC) is one of the most common malignant tumors of the digestive tract. According to the research of circular RNAs in the CRC field, compared with linear RNAs, circular RNAs are a special type of noncoding RNA that are covalently closed circular structures, which have no 5' cap structure and 3' polyA tail and are not affected by RNA exonuclease and actinomycin D.

**Biological functions:**

Notably, circular RNAs have a high degree of stability and potential effect on gene regulation. Meanwhile, circular RNAs are involved in the sponge action of microRNAs and mediate protein translation and direct binding, alternative splicing, and histone modification.

**Relationships with CRC:**

Studies have shown that circular RNAs are related to the proliferation, invasion, recurrence, metastasis, ferroptosis, apoptosis, and chemotherapy resistance of CRC.

**Conclusions:**

This article provides a brief review based on the source, structural characteristics, mechanisms, biological functions of circular RNAs, and the relationships between CRC.

## Graphical abstract

Studies have shown that circular RNAs are involved in the sponge action of microRNAs, mediate protein translation and direct binding, alternative splicing, histone modification, and their stability are much higher than linear RNAs. It is worth noting that circular RNAs are related to the proliferation, invasion, recurrence, metastasis, apoptosis, and chemotherapy resistance of colorectal cancer (CRC). Therefore, we summarized the relationship between the clinical value of circular RNAs and CRC, as well as the emerging trends and important areas of significant influence brought by circular RNAs.

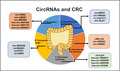

## 1 INTRODUCTION

Colorectal cancer (CRC) is one of the most common malignant tumors of the digestive tract, and morbidity and mortality rank third and second in malignant tumors.^1^, ^2^, ^3^ Currently, early screening for CRC can effectively improve the survival rate of patients with CRC, but approximately 25% of CRC patients develop metastatic disease from an early stage.^4^, ^5^, ^6^ Therefore, a promising field of antimetastatic therapy lies in the targeted inhibition of cancer cells with high metastatic potential from the primary site.^7^, ^8^ Recently, there has been a qualitative leap in tumor medical technology, such as the application of laparoscopic surgery, nano‐assembly technology, PET/CT imaging technology, and dynamic network biomarker (DNB), etc.^9^, ^10^, ^11^ However, the cure rate and survival rate of CRC are still very low, mainly lacking new drug treatment targets and biomarkers for the treatment of CRC.^12^ According to the research of circular RNAs in the CRC field, circular RNAs are expected to be therapeutic targets and biomarkers for CRC and promote great progress in the medical field of CRC treatment.^13^, ^14^, ^15^, ^16^

Circular RNAs (circRNAs) are a class of special noncoding RNA molecules formed from the 3’ to 5’ end rings of RNA that are highly expressed in the eukaryotic transcriptome.^17^, ^18^ Compared with linear RNAs, circRNAs have covalently closed circular structures without 5' cap structure and a 3' polyA tail and are much more stable than linear RNAs (with a half‐life beyond 48 h),^13^, ^17^, ^19^, ^20^ which have a high degree of stability and potential effect on gene regulation.^21^, ^22^, ^23^ In short, there is an urgent need to explore the source, classification, structural characteristics, and biological functions of circRNAs and a comprehensive understanding of the occurrence and development of CRC, chemotherapy resistance, recurrence and metastasis, and drug treatment targets and biomarkers.

## 2 THE ORIGIN, CLASSIFICATION, AND STRUCTURAL CHARACTERISTICS OF circRNAs

**FIGURE 1:**
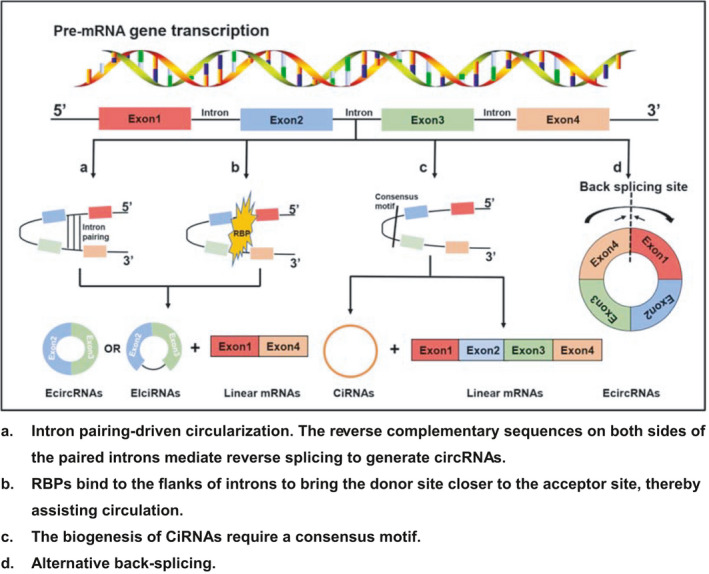
The biogenesis of circRNAs. (A) Intron pairing‐driven circularization. The reverse complementary sequences on both sides of the paired introns mediate reverse splicing to generate circRNAs. (B) RBPs bind to the flanks of introns to bring the donor site closer to the acceptor site, thereby assisting circulation. (C) The biogenesis of CiRNAs require a consensus motif. (D) Alternative back‐splicing

CircRNAs were first discovered in 1976; they were produced from an irregular reverse splicing sequence in pre‐mRNA with potential for gene regulation, and the upstream and downstream specific splicing sites are covalently linked and have tissue specificity.^15^, ^24^, ^25^, ^26^ Viroid was the first circRNA to be discovered, and in the following years, circRNAs in the nuclei of eukaryotes were found by electron microscopy.^19^, ^27^ With the continuous application of high‐throughput RNA sequencing technology, new circRNAs will continue to be discovered. Depending on the sequence of the genome, circRNAs are divided into three types: exonic circRNAs (EcircRNAs), exon–intron circRNAs (EIciRNAs), and intronic circRNAs (CiRNAs)^28^ (Figure 1). The circular structure and stability of circRNAs can be determined by RNA exonuclease (RNase), actinomycin D, and oligo (dT) primer. Among them, the oligo (dT) primer is a nucleotide chain consisting of thymine that binds to the polyA tail on the mRNA, notably, the binding amount of circRNAs and oligo (dT) primer is significantly less than linear RNAs. In addition, compared with linear RNAs with or without polyA tail, circRNAs can be resistant to RNase R and inhibit the transcription of actinomycin D. These results indicate that circRNAs have no 5' cap structure and 3' polyA tail, which are not easily degraded by RNase R, and under the activity of actinomycin D expression is very stable.^7^, ^21^, ^23^, ^29^ Recently, noncoding RNAs have played an important regulatory role in the field of cancer, among which circRNAs and microRNAs have a greater impact.^12^, ^30^ Compared with classic tumor markers such as CEA, CA‐125, and CA‐199, circRNAs and miRNAs have greater advantages in the diagnostic sensitivity and specificity of CRC.^31^, ^32^ Importantly, circRNAs are stably expressed in vivo and can serve as diagnostic markers for cancer, as well as various immune diseases.^33^, ^34^, ^35^ Meanwhile, circRNAs participate in acting as miRNA sponges, histone modification, protein translation and direct combination, alternative splicing, ferroptosis, and apoptosis. This indicates that they are suitable to be used as potential drug therapeutic targets and new clinical biomarkers in the treatment of CRC.^23^, ^36^, ^37^

## 3 MECHANISMS AND BIOLOGICAL FUNCTIONS OF circRNAs

### 3.1 CircRNAs act as miRNAs sponges in CRC

In terms of function, recent studies have confirmed that circRNAs contain many miRNA‐binding sites and act as miRNA sponges in CRC cells as competitive endogenous RNAs (ceRNAs) by binding to miRNAs and specific antagonism. This “sponging” is competitive inhibition.^17^, ^38^, ^39^, ^40^, ^41^ CircRNAs indirectly increase the expression levels of downstream target gene mRNAs and proteins by adsorbing miRNAs and participate in the occurrence and development of tumors.^42^, ^43^, ^44^, ^45^, ^46^ Xu et al. found that hsa‐circ‐000984 was mainly concentrated in the cytoplasm and identified the downstream target as miR‐106b, indicating that hsa‐circ‐000984 can act as a ceRNA by competitively binding miR‐106b, increasing the expression of cyclin‐dependent kinase 6 (CDK6) and participating in the proliferation and metastasis of CRC. It is worth noting that the high expression of hsa‐circ‐000984 is significantly related to TNM staging and the cell cycle. In addition, miR‐106b and the 3'UTR of CDK6 have complementary binding sites. Meanwhile, the abnormal expression of CDK4/6 may be an important sign in the field of CRC and bladder cancer, indicating that hsa‐circ‐000984 may have broad application prospects in the emerging field of molecular markers, which may be a breakthrough in targeted diagnosis of CRC.^38^, ^47^ At the same time, Shang et al. confirmed that circPACRGL and miR‐142‐3p/miR‐506‐3p have a specific spongy effect, thereby promoting the expression of transforming growth factor‐β1 (TGF‐β1). The TGF‐β family regulates CRC cell proliferation, differentiation, tumor angiogenesis, tumor immunity, and inflammatory response.^48^, ^49^, ^50^, ^51^, ^52^ This indicates that circPACRGL and TGF‐β1 have a coordinated effect and promote the differentiation of neutrophils from N1 to N2, which may lead to the occurrence of tumors. However, it is worth thinking about how circPACRGL in tumor‐derived exosomes regulates miRNAs and TGF‐β1 to affect CRC. Therefore, we conclude that the circPACRGL‐miR‐142‐3p/miR‐506‐3p‐TGF‐β1 axis is a breakthrough discovery in the treatment of CRC.^53^ As one of the most extensive circRNAs, circ‐CDR1as is formed by reverse transcription of the cerebellar denature‐associated antigen 1 (CDR1) gene, indicating that the pre‐linear RNA of CDR1as transcribed from the antisense strand of CDR1 will undergo 5' and 3' reverse splicing at the same time to form a circRNA, which is approximately 1485 bp in length and contains more than 70 miR‐7‐binding sites. Notably, miR‐7 can act as a tumor suppressor to induce growth factor signal transduction; therefore, we conclude that circ‐CDR1as may regulate the stability of the target gene miR‐7 through the sponge action of miRNAs, regulating insulin secretion and tumorigenesis.^39^, ^54^, ^55^ These results suggest that circRNAs can act as sponges for miRNAs and participate in the tumorigenesis of CRC, which is expected to be a breakthrough in the diagnosis and treatment of CRC.

### 3.2 CircRNAs are involved in protein translation

CircRNAs can be involved in protein translation, in which translation initiation elements and open reading frames play crucial roles.^28^ As an oncogene, hsa‐circ‐002144 can bind to miR‐615‐5p and colocalize with the cytoplasm of cancer cells. In addition, the target proteins of miR‐615‐5p, LARP1, and mTOR may serve as translation regulators, indicating that overexpression of them can rescue the effect of hsa‐circ‐002144 knockdown on tumors. Meanwhile, inhibition of miR‐615‐5p can restore the inhibitory effect of hsa‐circ‐002144 on LARP1 and mTOR proteins. It is worth noting that miR‐615‐5p has been reported to be involved in tumor angiogenesis, and the mTOR protein regulated by the phosphatidylinositol 3‐kinase/Akt pathway is also involved in the proliferation and angiogenesis of CRC, which indirectly indicates that its mechanism may be related to tumor angiogenesis and protein translation. Therefore, we conclude that hsa‐circ‐002144, LARP1, and mTOR proteins may have synergistic stimulatory effects on protein translation, providing an effective target for future clinical treatment of CRC. However, it is worth thinking about whether the phosphatidylinositol 3‐kinase/Akt pathway regulates hsa‐circ‐002144 for tumor intervention; furthermore, how hsa‐circ‐002144 regulates E‐cadherin and N‐cadherin and participates in the epithelial–mesenchymal transition of CRC needs to be verified.^25^ Hsa‐circ‐0001492 circulates in exons 2–4 of the ERBIN gene, called circ‐ERBIN, and is mainly located in the cytoplasm. Meanwhile, circ‐ERBIN can also activate hypoxia‐inducible factor‐1α (HIF‐1α) to improve tumor angiogenesis, indicating that circ‐ERBIN coregulates the translation of 4E binding protein 1 (4EBP‐1) to increase the protein level of HIF‐1α and promote the phosphorylation state through mTOR signaling at the non‐cap‐dependent protein level. It is noteworthy that ERBIN is involved in a variety of malignant tumors, including CRC, but linear ERBIN is easily degraded under the action of actinomycin D. Among them, ERBIN maintains cell polarity, indicating that circ‐ERBIN and ERBIN have a common trend in CRC tumorigenesis and protein translation; however, the regulatory mechanism of circ‐ERBIN and linear ERBIN is not yet clear. Therefore, we conclude that circ‐ERBIN may maintain the activity of 4EBP‐1, suggesting that circ‐ERBIN may be a promising target for CRC treatment.^56^ Hsa‐circ‐02276, its peptide, can bind to Lgr4, called circLgr4, which is highly expressed in CRC and is mainly concentrated in the nucleus. Notably, circLgr4 has peptide coding ability and participates in protein coding and regulation in a peptide‐dependent manner, indicating that the peptide encoded by circLgr4 can be combined with Lgr4 to mediate the proliferation and metastasis of CRC.^57^ These results indicate that circRNAs can participate in protein translation and maintain the growth of intestinal cancer cells, providing a promising target for future clinical treatment of CRC.

### 3.3 CircRNAs interact with RNA‐binding proteins (RBPs)

CircRNAs interact with RNA‐binding proteins by adsorbing proteins. RNA‐binding proteins have complete RNA‐binding domains that bind double‐stranded or single‐stranded RNA and are involved in the biological processes of RNA, such as RNA transcription, RNA modification, pre‐mRNA splicing, and RNA localization. In addition, some RBPs are also involved in the biogenesis of circRNAs.^58^, ^59^ Hsa‐circ‐0008558 is composed of LONP2 exons 8–11, called circLONP2, and is mainly distributed in the nucleus. It is worth noting that circLONP2 cooperates with FUS and DDX1 to form a complex, which may promote the maturation of miR‐17‐5p by recruiting DGCR8/Drosha protein, thereby playing the role of a key metastasis‐initiating molecule in CRC. Among them, FUS is an RNA‐binding protein that regulates circRNAs in mouse embryonic stem cells and regulates mouse motor neurons, which also activates the transcription of X‐linked apoptosis inhibitor protein (XIAP) and mediates tumorigenesis as a key regulatory factor.^60^, ^61^ However, it is worth thinking about the mechanism of how miR‐17‐5p is assembled into exosomes and how circLONP2 participates in regulation. Therefore, we conclude that circLONP2 can be used as an effective antimetastatic therapeutic target, which may be a breakthrough in CRC antimetastatic treatment.^7^ CircZNF609 participates in protein coding; furthermore, FUS can also bind to the pre‐mRNA of ZNF609 to increase circZNF609 expression. However, more evidence is needed to confirm that FUS can promote the circulation of circRNAs. This suggests that under the combination of G protein subunit β2 (GNB2) and FUS, circZNF609 may be a breakthrough in the detection and treatment of liver cancer research.^62^ Hsa‐circ‐0008367 is derived from the 13th and 14th exons of the IARS gene and is called circ‐IARS. It is worth noting that m6A methylation is the most abundant posttranscriptional mRNA methylation in eukaryotes and is one of the most common RNA modifications, including the processing of primary miRNAs, the maturation of miRNAs, and the interaction of RNA‐binding proteins. Among them, ALKBH5 was reported to be an N6‐methyladenosine (m6A) eraser. However, ALKBH5 also acts as an RBP and cooperates with circ‐IARS to regulate ferritin autophagy, indicating that circ‐IARS is dependent on the negative regulation of the autophagy inhibitor ALKBH5 to participate in autophagy. Finally, we conclude that circ‐IARS can significantly inhibit autophagy and ferric phagocytosis of ALKBH5 by interacting with the m6A demethylase ALKBH5.^27^, ^63^, ^64^, ^65^, ^66^ These results indicate that circRNAs may interact with RNA‐binding proteins by adsorbing proteins to participate in the progression of CRC.

### 3.4 circRNAs and alternative splicing

Alternative splicing (AS) of RNA refers to the splicing of pre‐mRNA at different splicing sites, selectively removing introns and retaining exons, thereby generating two or more mRNA transcripts to increase protein diversity. AS mainly relies on splice bodies, splice sites, cis‐acting elements, and trans‐acting factors to participate in the proliferation, invasion, chemotherapy resistance, and apoptosis of various cancers.^67^ A variety of circRNAs containing exons or introns can be generated by a single gene through alternative splicing. A large number of studies have found that circRNAs are covalently linked to upstream and downstream‐specific splicing sites generated from standardized sequences in pre‐mRNA or nonstandardized reverse splicing sequences that have potential for gene regulation.^24^, ^25^, ^26^ Most circRNAs are exon circRNAs, which are produced by reverse splicing of exon regions of known protein‐coding genes.^68^, ^69^, ^70^ Hsa‐circ‐000984 is located at 7q of the chromosome region, which contains an exon formed by head‐to‐tail splicing.^38^ Circ‐ERBIN circulates in exons 2–4 of the ERBIN gene, indicating that circ‐ERBIN is highly conserved and regulates the translation of 4E binding protein 1 (4EBP‐1) in the transcriptome, increasing the protein level of HIF‐1α.^56^ Hsa‐circ‐0008558 is composed of LONP2 exon 8–11, originating in a specific reverse splicing sequence and a specific back‐junction site of circLONP2. It is worth noting that circLONP2 can coordinate with DDR1 (Discoidin domain receptor 1) to regulate the maturation of pri‐miR‐17, indicating that it may drive mature miR‐17‐5p to promote the tumorigenesis of CRC.^7^, ^71^ CircRNAs compete with each other for circularization and splicing, indicating that EcircRNAs may play an important role in AS. Reverse splicing usually creates a specific dorsal splicing site to splice internal exons to form AS. At the same time, EcircRNAs can also block the translation initiation site and generate a large number of noncoding linear transcripts, thereby reducing the protein level of mRNA. It is worth noting that EIciRNA may also be separated during this process, and the truncated linear mRNA cannot participate in protein translation.^72^, ^73^, ^74^, ^75^ Therefore, we conclude that similar to linear RNAs, circRNAs produced by reverse splicing of exons may play significant regulatory roles in the transcriptome through alternative splicing and participate in the occurrence and development of tumors.^76^, ^77^

### 3.5 CircRNAs participate in histone modification

**FIGURE 2:**
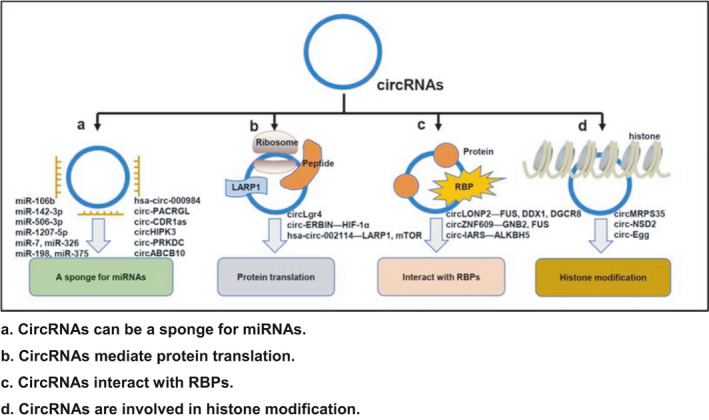
Mechanisms and biological functions of circRNAs. (A) CircRNAs can be a sponge for miRNAs. (B) CircRNAs mediate protein translation. (C) CircRNAs interact with RBPs. (D) CircRNAs are involved in histone modification

Histone modifications include methylation, acetylation, glycosylation, phosphorylation, and ubiquitination.^78^ In many eukaryotes, methylation of the 5' end of mRNA plays a significant biological role in mRNA splicing, degradation, and stability. Methylation of the 3' terminal contributes to the transport of the mRNA and maintains the stability of the mRNA together with the polyA‐binding protein.^63^, ^79^ Hsa‐circ‐0000384 is derived from exons 2, 3, 4, and 5 of the MRPS35 gene, called circMRPS35, and is mainly concentrated in the nucleus. As a tumor suppressor gene, circMRPS35 has a negative correlation with lymph node metastasis and TNM staging. Notably, lysine acetyltransferase 7 (KAT7) is more prone to lysine 5, lysine 12, and acetylated H4. Moreover, as the coordination partner of KAT7, circMRPS35 may significantly increase the H4K5 acetylation level of the target genes FOXO1 and FOXO3a. Therefore, we conclude that circMRPS35 combined with KAT7 acetylation may mediate FOXO1 and FOXO3a to interfere with tumor metastasis, laying a solid foundation for tumor intervention, which may be a major breakthrough in regulating tumors at the level of histone acetylation.^23^ Chen et al. screened CRC transfer‐related circ‐NSD2 from NSD2 exons 1–2 as a histone methyltransferase in a CLM mouse model and confirmed that circ‐NSD2 was mainly concentrated in the cytoplasm and partially concentrated in the nucleus. It is worth noting that circ‐NSD2 can activate DDR1 and JAG1 (Jagged1) genes. Among them, DDR1 and JAG1, either alone or in combination, may participate in CRC migration and proliferation, indicating that overexpression of DDR1 and JAG1 can effectively reverse the effect of circ‐NSD2 knockdown on CRC, providing a diagnostic target for the treatment of CRC liver metastasis.^18^ CircEgg adsorbs miR‐3391‐5p through acting as a sponge, inhibits histone H3 lysine 9 methylation (H3K9me3), promotes histone H3 lysine 9 acetylation (H3K9ac), and positively regulates histone deacetylase (HDAC) Rpd3 gene expression. It is worth noting that circEgg can encode the circEgg‐P122 protein to inhibit the methylation of H3K9me3. These results suggest that circEgg may interact with miR‐3391‐5p and the circEgg‐P122 protein encoded by miR‐3391‐5p to regulate histone modification, among them, methylation is transcriptional inhibition, and acetylation is transcriptional activation, which are the main epigenetic histone modification, thereby regulating the genome‐wide expression of genes. In addition, this is the first time that the histone modification of circRNA has played an important role in epigenetics, indicating that in the diagnosis of CRC and other cancers, epigenetics combined with histone modification may make a major breakthrough. However, it is worth considering how m6A methylation participates in this regulatory mechanism, which still needs to be explored^80^ (Figure 2).

## 4 CircRNAs AND CRC

### 4.1 CircRNAs mediate the occurrence and development of CRC

CircRNAs can regulate the maturation of miRNAs, and miRNAs can regulate the mRNA levels of target genes. That is, through the interaction of circRNAs–miRNAs and miRNAs–mRNAs, a circRNAs–miRNAs–mRNAs regulatory network is formed to participate in the occurrence and development of CRC.^81^, ^82^, ^83^, ^84^ Hsa‐circ‐0000284 is derived from exon 2 of linear HIPK3, called circHIPK3, which is highly expressed in CRC and mainly expressed in the cytoplasm. Therefore, we conclude that circHIPK3 may play a positive regulatory role in the tumorigenesis of CRC by sponging miR‐1207‐5p, which directly targets FMNL2. It is worth noting that overexpression of FMNL2 significantly increased the levels of p‐IKKα/β, p‐IκBα, and p65, thus activating the NF‐κB signaling pathway. On the other hand, the Rho GTPase pathway, Wnt pathway, G‐protein pathway, and P53 pathway were affected following FMNL2 knockdown, indicating that circHIPK3/miR‐1207‐5p/FMNL2 orchestrating multiple pathways may mediate the tumorigenesis of CRC.^85^, ^86^, ^87^ JIN et al. confirmed that the protein kinase catalytic subunit (PRKDC) hsa‐circ‐0136666, called circ‐PRKDC, is highly expressed in CRC.^88^ On this basis, circ‐PRKDC can target miR‐198 through the sponge action of miR‐198 and promote the expression of DDR1 and FOXM1 mRNA and protein, indicating that inhibition of miR‐375 or overexpression of FOXM1 can effectively increase the reduction in β‐catenin and c‐Myc protein levels in the Wnt/β‐catenin pathway caused by knockdown of circ‐PRKDC. Therefore, we conclude that circ‐PRKDC and FOXM1 may have a synergistic effect on tumor growth, thereby participating in the tumorigenesis of CRC by regulating the miR‐198/DDR1 axis or in combination with miR‐375/FOXM1 and the Wnt/β‐catenin pathway.^89^, ^90^ Notably, ferroptosis is an iron‐dependent regulatory cell death mode discovered in recent years that is mainly manifested by disorder of cellular iron metabolism and induction of mitochondrial lipid peroxides. However, the involvement of circRNAs in ferroptosis can also effectively affect the occurrence and development of CRC.^91^, ^92^ XIAN et al. confirmed that circABCB10 mainly interacts with miR‐326 and C‐C motif chemokine ligand 5 (CCL5), suggesting that knockdown of miR‐326 can target CCL5 to alleviate ferroptosis and apoptosis of CRC cells caused by circABCB10 knockdown. Therefore, we conclude that circABCB10 may participate in the sponge regulation of miR‐326 to regulate CCL5 and ultimately promote the ferroptosis and apoptosis of CRC cells, so ferroptosis regulated by circABCB10 is expected to be a major breakthrough in the occurrence and development of CRC.^93^

### 4.2 circRNAs participate in clinical chemotherapy resistance of CRC

CircRNAs are involved in metastasis and invasion of CRC, epithelial–mesenchymal cell transformation, blood vessel formation, immune response, immune escape, and tumor chemotherapy resistance.^32^, ^53^, ^94^ Malignant tumors often produce ATP through aerobic oxidation of glycolysis, thus guaranteeing the growth of CRC and resistance to chemotherapy.^95^ The M2 subtype of pyruvate kinase (PKM2) is the main type of cancer cell proliferation, which indicates that when PKM2 is overexpressed, it may increase the rate of glycolysis and produce a large amount of ATP, thus promoting tumor growth and chemotherapy resistance.^96^ It is worth noting that oxaliplatin is the main treatment for CRC; however, oxaliplatin resistance has emerged during the treatment of CRC. WANG et al. demonstrated that exosomes from oxaliplatin‐resistant CRC cells were delivered to monolayer‐sensitive cells via the transport of hsa‐circ‐0005963, which interacted with miR‐122 through two potential binding regions, called ciRS‐122. Therefore, we conclude that ciRS‐122 in serum exosomes of CRC patients may target the miR‐122 sponge to upregulate PKM2 and promote glycolysis and chemotherapy resistance in tumors and increase ATP production, indirectly showing that they are important participants in the tumorigenesis of CRC.^97^ XU et al. detected circ‐FBXW7 (circRNA F‐box and containing a 7 WD repeat domain) in exosomes, which is mainly expressed in the cytoplasm. Notably, circ‐FBXW7 had low expression in cells or tissues of oxaliplatin‐resistant CRC patients, indicating that circ‐FBXW7 in exosomes can induce the sensitivity of CRC cells to oxaliplatin, induce apoptosis, and inhibit the transformation of epithelial–mesenchymal cells. However, it is worth thinking that Wnts derived from exosomal carcinoma‐associated fibroblasts can inhibit CRC chemosensitivity, which requires further investigation. Therefore, we conclude that the combination of circ‐FBXW7 delivered by exosomes directly with its target miR‐18b‐5p may improve the sensitivity of CRC cells to oxaliplatin and chemotherapeutic resistance, suggesting that circ‐FBXW7 delivered by exosomes has made a major breakthrough in the field of oxaliplatin clinical tumor chemotherapy resistance.^98^, ^99^

### 4.3 CircRNAs are involved in CRC recurrence and metastasis

According to reports, the process of tumor metastasis is multi‐step, including invasion, infiltration, blood circulation, extravasation, and colonization.^7^ A serious problem in the treatment of CRC is the recurrence and metastasis of cancer, which are all due to the presence of cancer stem cells (CSCs) in the tumor. For circRNAs in the spherical cells of CRC rich in CSCs, circRNAs can be involved in the recurrence and metastasis of CRC by mediating the regulatory network of circRNAs–miRNAs–mRNAs. Like most malignant tumors, the metastasis of CRC is a complex biological process involving multiple signaling pathways and multiple mechanisms. For example, the expression levels of hsa‐circ‐0066631 and hsa‐circ‐0082096 in CRC spherical cells were significantly upregulated, mainly concentrated in the nucleus. In addition, they can target the sponging effect of miRNAs to downregulate miR‐140‐3p, miR‐224, miR‐382, miR‐548c‐3p, and miR‐579. Not only that, mRNA encoding proteins such as ACVR1C/ALK7, FZD3, IL6ST/GP130, SKIL/ SNON, SMAD2, and WNT5 are further regulated. This result indicates that the encoded proteins may activate TGF‐β/SMAD, Wnt/β‐catenin, and other signaling pathways to participate in CRC recurrence and metastasis.^100^ CircLgr4 is highly expressed in CRC stem cells and may interact extensively with other molecules, including microRNA, chromatin remodeling complex, and RNA polymerase II. Finally, we conclude that circLgr4 may mediate self‐renewal of CSCs in CRC for recurrence and metastasis, indicating that circLgr4 may have a positive effect on tumor growth.^57^ Hsa‐circ‐0000598 is composed of the B2M gene on chromosome 15, also known as hsa‐circ‐001680, which is highly expressed in CRC globular cells. Among them, hsa‐circ‐001680 was mainly concentrated in the cytoplasm and enhanced the expression of downstream target gene BMI1 by inhibiting miR‐340 with sponges. Therefore, we conclude that hsa‐circ‐001680 and BMI1 may play a coordinated role in the recurrence and metastasis of tumors, indicating that knockdown of hsa‐circ‐001680 could reverse the overexpression of BMI1 caused by knockdown of miR‐340.^101^, ^102^ It is worth noting that hsa‐circ‐001680 can increase the proportion of CRC stem cells and increase the expression of stem cell markers such as SOX2, CD44, and CD133 from mRNA and protein levels, in which cancer cells with surface markers CD44+/CD133+ can develop their stem cell properties. However, CRC stem cells also have the function of regulating drug resistance to chemotherapy, indicating that hsa‐circ‐001680 may induce drug resistance of CRC to irinotecan and reduce apoptosis of CRC cells by upregulating the expression of stem cells. The results show that breakthroughs have been made in the development of new irinotecan‐resistant treatment strategies. Therefore, circRNA enriched in cancer stem cells will have a significant impact in the field of CRC recurrence, metastasis, and irinotecan chemotherapy resistance.^101^, ^103^

### 4.4 CircRNAs can be potential therapeutic targets for CRC

As an important tumor regulator in CRC, circRNAs are expected to become potential drug treatment targets for CRC. Circ‐0000392 was originated from exons 2–4 of the YAF2 gene, with a splicing length of approximately 326 bp, mainly located in the cytoplasm. For the mechanism, we conclude that circ‐0000392 further regulates the expression of PIK3R3 through acting as a sponge of miR‐193a‐5p and thereby may affect the AKT‐mTOR pathway in the CRC tumor microenvironment.^104^ This result indicates that knocking down miR‐193a‐5p can alleviate the reduction of PIK3R3 mRNA and protein levels and the reduction of AKT and mTOR phosphorylation levels caused by knocking down circ‐0000392. Among them, the mTOR protein can regulate the phosphatidylinositol 3‐kinase. However, abnormal activation of the AKT‐mTOR siganling pathway may indicate the malignant occurrence of CRC. Therefore, it can be seen that circ‐0000392 may become a potential clinical treatment target for CRC.^13^ CircRUNX1 was derived from exons 2 and 3 of the RUNX1 gene, with a base sequence length of approximately 297 bp, and it is mainly located in the cytoplasm. As an oncogene, circRUNX1 was positively correlated with tumor staging, lymph node metastasis, and distant metastasis in CRC patients. It is worth noting that circRUNX1 can competitively bind to miR‐145‐5p to upregulate IGF1. Among them, the insulin‐like growth factor (IGF) plays an important regulatory role in CRC and other malignant tumors, indicating that circRUNX1 can be used as a tumor promoter and is expected to become a potential drug treatment target for CRC.^105^, ^106^, ^107^, ^108^, ^109^ In CRC, circPVT1 is upregulated and participates in the proliferation and metastasis of CRC. However, it is noteworthy that overexpression of miR‐145 significantly reverses the effect of upregulated circPVT1 on the metastasis of CRC, suggesting that miR‐145 is a key downstream effector of circPVT1‐mediated CRC metastasis. Therefore, we conclude that the circPVT1/miR‐145 axis may become a new drug therapy target for the treatment of CRC.^37^ Hsa‐circ‐0006990 (circVAPA) is highly expressed in CRC tissues, mainly in the cytoplasm. Not only that, circVAPA can also be detected in plasma and saliva, indicating that it may become a promising biomarker for CRC. In addition, the upregulation of miR‐101 can significantly inhibit the effect of overexpression of circVAPA on CRC, suggesting that circVAPA engulfed miR‐101. However, it is worth noting that circVAPA may also mediate CRC by regulating the expression of VAPA, among them, the VAPA can act as a ceRNA to regulate phosphatase and tensin homolog (PTEN) to play a tumor suppressor effect. However, it is worth considering that the joint regulation mechanism of circVAPA and VAPA needs to be further explored. Therefore, we conclude that circVAPA may promote the progress of CRC and is expected to become a potential drug target.^14^

### 4.5 CircRNAs can be used as biomarkers for CRC treatment

**FIGURE 3:**
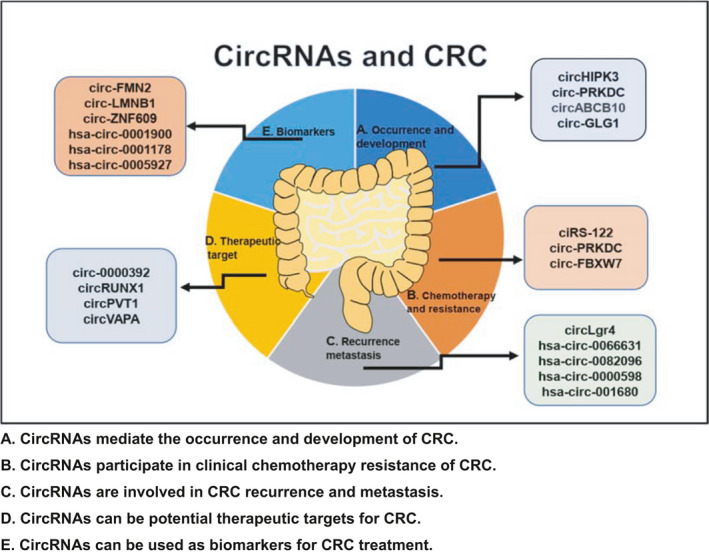
The relationships of circRNAs and CRC. (A) CircRNAs mediate the occurrence and development of CRC. (B) CircRNAs participate in clinical chemotherapy resistance of CRC. (C) CircRNAs are involved in CRC recurrence and metastasis. (D) CircRNAs can be potential therapeutic targets for CRC. (E) CircRNAs can be used as biomarkers for CRC treatment

At present, although imaging examination and endoscopy are relatively common and reliable methods for the detection of CRC, the accuracy of screening is often reduced due to poor patient compliance, high price, and intrusive characteristics, so it is not suitable for the examination of critically ill patients. In addition, compared with noninvasive biomarkers for CRC screening, including CEA and fecal occlusive blood test (FOBT), circRNAs have higher accuracy, sensitivity, and specificity, indicating that they can often be used as biomarkers for the diagnosis and treatment of CRC.^16^, ^110^, ^111^, ^112^, ^113^ ZHANG et al. detected the expression levels of circRNAs in the serum of CRC patients and healthy people. It is concluded that the expression levels of circ‐FMN2, circ‐LMNB1, and circ‐ZNF609 were higher than those of the healthy control group, their expression was positively correlated with histological grade, lymph node metastasis, and TNM staging, suggesting that circ‐FMN2, circ‐LMNB1, and circ‐ZNF609 are likely to be biomarkers for the diagnosis of CRC.^16^ For the quality control of circRNAs and miRNAs in serum or plasma samples, it is worth noting here that the hemolyzed sample has little effect on the concentration of circRNAs and miRNAs, and there is no correlation. In addition, plasma or serum samples are separated from peripheral blood and transferred to a 1.5 ml RNase‐free tube, stored in liquid nitrogen, without repeated freezing and thawing, which truly reflect the expression of circRNAs and miRNAs in vivo.^16^, ^114^, ^115^, ^116^, ^117^, ^118^ LI et al. screened out specific circRNAs in plasma of CRC patients, evaluated the clinical value of circRNAs in plasma, finally determined that the expression of hsa‐circ‐0001900, hsa‐circ‐0001178, and hsa‐circ‐0005927 in plasma of CRC patients was upregulated relative to that of healthy controls, indicating that these circRNAs are expected to be used as biomarkers for CRC. It is worth noting that the combination of circRNAs and CEA has a higher diagnostic accuracy rate than CEA. Therefore, we conclude that circRNAs can significantly increase the sensitivity and specificity of diagnosis by combining classic biomarkers, indicating that co‐diagnosis is expected to be a key breakthrough in the progression of CRC and other tumors.^110^ HAO et al. screened out a highly expressed hsa‐circ‐0003315 in CRC from Golgi glycoprotein 1 mRNA, with a length of approximately 477 nucleotides, which was formed by reverse splicing of exon 5–8 of GLG1 gene, called circ‐GLG1. In addition, circ‐GLG1 can absorb miR‐622, regulating the expression of KRAS at the mRNA and protein levels. However, it is worth noting that by inhibiting miR‐622, the decrease in KRAS mRNA and protein levels caused by knocking down circ‐GLG1 can be reversed. Meanwhile, the suppression of CRC proliferation and invasion can also be alleviated. This result indicates that circ‐GLG1 and KRAS may synergistically promote the invasion and metastasis of CRC and are expected to become biomarkers involved in the diagnosis and treatment of CRC^119^ (Figure 3; Table 1).

**TABLE 1:** Source, localization, mechanism, and biological function of circRNAs

CircRNAs	Location	Mechanisms of action	Molecule/axe/signaling pathway	Biological function	Reference
Hsa‐circ‐0001492(circ‐ERBIN)	Cytoplasm	HIF‐1α was upregulated by targeting miR‐125a‐5p and miR‐138‐5p sponging	miR‐125a‐5p/miR‐138‐5p/4EBP‐1	Carcinogenic function, promote tumor angiogenesis, and increase the level of HIF‐1α protein	[56]
Hsa‐circ‐0069313(circ‐PACRGL)	Cytoplasm	As the sponges of miR‐142‐3p and miR‐506‐3p	miR‐142‐3p/miR‐506‐3p/TGF‐β1	Carcinogenic function, promote the expression of TGF‐β1, participate in tumor immune response, and promote the differentiation of N1–N2 neutrophils	[53]
Circ‐CDR1as	Cytoplasm	The stability of target gene miR‐7 was regulated through the sponging effect of miRNAs	miR‐7/HOXB13	Carcinogenic function, regulate insulin secretion, promote CRC cell proliferation, and differentiation and tumor metastasis	[55]
Hsa‐circ‐0008558(circ‐LONP2)	Nucleus	Under FUS regulation, pre‐mLONP2 can be transformed into circLONP2, which recruits DGCR8 in a DDX1‐dependent manner and promotes the maturation of miR‐17‐5p	Not applicable	Carcinogenic function, driving mature miR‐17‐5p to bind to exosomes has high metastasis potential, and promoting the proliferation and metastasis of CRC	[7]
Hsa‐circ‐0003315(circ‐GLG1)	Cytoplasm	As a sponge of miR‐622, the length is about 477 nucleotides	miR‐622/KRAS	Carcinogenic function, promote the proliferation and metastasis of CRC, which can be used as biomarkers for clinical diagnosis of CRC	[119]
Hsa‐circ‐0000384(circMRPS35)	Nucleus	Recruit KAT7 to attach to the promoters of FOXO1 and FOXO3a to increase the level of H4K5 acetylation	KAT7/H4K5/FOXO1/3a	Antitumor effect, negatively related to tumor lymph node metastasis, TNM staging, and tumor size	[23]
Circ‐NSD2	Cytoplasm (main), nucleus	As a sponge of miR‐199b‐5p, activated DDR1 and JAG1 genes are involved in histone modification	miR‐199b‐5p/DDR1/JAG1	Oncogenic function, as a histone methyltransferase coordinator, helps CRC cell matrix interaction, migration, and metastasis, providing a diagnostic target for the treatment of CRC liver metastasis	[18]
Hsa‐circ‐002144	Cytoplasm	As a sponge for miR‐615‐5p	miR‐615‐5p/LARP1	Carcinogenic function, regulate LARP1 to promote the progression of colorectal cancer, and provide a therapeutic target for tumor intervention	[25]
Hsa‐circ‐0000677 (circABCB10)	Cytoplasm	CircABCB10 targets sponge miR‐326 and interacts with CCL5	miR‐326/CCL5	Knockdown of cirABCB10 can promote iron death and apoptosis of CRC cells and inhibit the proliferation and metastasis of CRC	[93]
Hsa‐circ‐0008367(circ‐IARS)	Cytoplasm	The interaction of circ‐IARS with RBP (ALKBH5) inhibited the autophagy and ferritin phagocytosis of ALKBH5	circ‐IARS/ALKBH5	Carcinogenic function, induces iron death to participate in tumor proliferation and metastasis	[27]
Hsa‐circ‐0005963(ciRS‐122)	Nucleus	Sponge action targeting miR‐122 via PKM2	miR‐122 /PKM2	It promotes the aerobic oxidation of the glycolysis of CRC and produces a large amount of ATP, which in turn promotes the growth of CRC and the resistance to chemotherapy	[97]
Circ‐FBXW7	Cytoplasm	Circ‐FBXW7 is a sponge for miR‐18b‐5p in CRC cells and is negatively correlated with miR‐18b‐5p	circ‐FBXW7/miR‐18b‐5p	Antitumor effect, increase the chemotherapy resistance of oxaliplatin in CRC cells	[98]
Hsa‐circ‐000984	Cytoplasm	Competitive binding of miR‐106b exerts the role of ceRNA and upregulates the expression of CDK6	miR‐106b/CDK6	The carcinogenic effect was significantly correlated with advanced T`NM (stage 3 + stage 4)	[38]
Hsa‐circ‐0000284(circHIPK3)	Cytoplasm	CircHIPK3 regulates FMNL2 through the sponge action of miR‐1207‐5p	miR‐1207‐5p/FMNL2	Carcinogenic effect, promote the proliferation, and invasion and metastasis of CRC	[87]
Hsa‐circ‐0066631, hsa‐circ‐0082096	Nucleus	Sponge targeting of miRNAs, such as miR‐140‐3p, miR‐224, miR‐382, miR‐548c‐3p, and miR‐579	ACVR1C/ALK7, FZD3, IL6ST/GP130, SKIL/SNON, SMAD2, WNT5; TGF‐β/SMAD, Wnt/β‐catenin	Rich in cancer stem cells, involved in the recurrence and metastasis of CRC	[100]
CircRNA‐0000392	Cytoplasm	The expression of PIK3R3 was regulated by the sponges of miR‐193a‐5p	miR‐193a‐5p/PIK3R3/AKT	The carcinogenic effect promotes the proliferation and invasion of CRC, which can be used as therapeutic targets and biomarkers for colorectal cancer	[13]
CircRUNX1	Cytoplasm	CircRUNX1 acts as a sponge of miR‐145‐5p to upregulate IGF1	miR‐145‐5p/IGF1	As a tumor promoter, it is involved in the proliferation, invasion, cell cycle progression, and apoptosis of CRC cells	[105]
Hsa‐circ‐0006990(circVAPA)	Cytoplasm	CircVAPA targeting sponge miR‐101‐3p	Not applicable	Significantly promote the proliferation, migration, and metastasis of CRC, and participate in the cell cycle process; Inhibit cell apoptosis, which is expected to become a potential therapeutic target for CRC	[14]
Hsa‐circ‐0136666(circ‐PRKDC)	Cytoplasm	Circ‐PRKDC promotes DDR1 mRNA and protein expression in CRC tissues by targeting miR‐198 through the sponging effect of miR‐198	miR‐198 /DDR1	Carcinogenic function, promote the proliferation, and invasion and metastasis of CRC	[89]
Hsa‐circ‐0000598(circ‐001680)	Cytoplasm	Circ‐001680 enhanced BMI1 expression by spongy inhibition of miR‐340. At the same time, BMI1 also regulates the stem cell‐like properties of cancer	miR‐340/BMI1	Carcinogenesis, which is related to clinical T staging of CRC patients, induces resistance of CRC to irinotecan, reduces apoptosis of colorectal cancer cells, and participates in recurrence and metastasis of CRC	[101]
Has‐circ‐02276(circLgr4)	Nucleus	Peptide coding ability, involved in protein coding and regulation in a peptide‐dependent manner	Peptide/Lgr4	Carcinogenesis, encoding proteins, and participating in CSC self‐renewal, tumor recurrence, and metastasis in colorectal cancer	[57]

Abbreviations: 4EBP‐1, 4E binding protein 1; CCL5, C‐C motif chemokine ligand 5; CDK6, cyclin‐dependent kinase 6; CRC, colorectal cancer; HIF‐1α, Hypoxia‐inducible factor‐1α; IGF1, insulin‐like growth factor 1; PKM2, the M2 subtype of pyruvate kinase; TGF‐β1, transforming growth factor‐β1.

## 5 CONCLUSION AND PERSPECTIVE

At present, although the clinical treatment effect of CRC has improved, due to its poor prognosis and high recurrence rate and metastasis rate, morbidity and mortality are still increasing year by year. In 2020, approximately 1.9 million new CRC cases and 935,000 CRC‐related deaths were reported globally, accounting for 10% of the global cancer morbidity rate and 9% of the mortality rate. According to the prediction of the development of human diseases, the global population of CRC cases will reach more than 3 million in the next 20 years.^120^, ^121^ Therefore, the key to the treatment of CRC is early detection, early diagnosis, and early treatment. As special noncoding RNA molecules in eukaryotes, circRNAs are formed by irregular reverse splicing sequences in pre‐mRNA through specific upstream and downstream splicing sites. Compared with linear RNAs, circRNAs have no 5' cap structure and 3' polyA tail and are not affected by RNA exonuclease and actinomycin D, which are highly conserved, widely distributed in human cells and have gene regulation potential. To a large extent, circRNAs are better than linear RNAs, indicating that circRNAs may play important roles in tumorigenesis, invasion, metastasis, and other processes and can become drug targets and clinical biomarkers for cancer treatment. However, due to their low abundance, circRNAs have not attracted wide attention. Currently, with the introduction and improvement of high‐throughput sequencing technology, new circRNAs will continue to be discovered and have a significant impact in many areas. The specific details are as follows: hsa‐circ‐000984 and circLONP2 have broad application prospects in the emerging field of molecular markers in the antimetastasis therapy of CRC. Meanwhile, circMRPS35 lays a solid foundation for tumor intervention, which may be a major breakthrough in regulating tumors at the level of histone acetylation. In addition, circEgg is the first time that the histone modification of circRNA has played an important role in epigenetics, indicating that in the diagnosis of CRC and other cancers, epigenetics combined with histone modification may make a major breakthrough. It is worth noting that ferroptosis regulated by circABCB10 is expected to be a major breakthrough in the occurrence and development of CRC. Moreover, circ‐FBXW7 delivered by exosomes has made a breakthrough in the field of oxaliplatin clinical tumor chemotherapy resistance, and circRNAs enrichment in cancer stem cells will have a significant impact in the field of CRC recurrence, metastasis, and irinotecan chemotherapy resistance. Furthermore, circRNAs can significantly increase the sensitivity and specificity of diagnosis by combining classic biomarkers, indicating that codiagnosis is expected to be a key breakthrough in the progression of CRC and other tumors. However, in the clinical immunotherapy and interventional therapy of tumors, the mechanism of action and biological function of exosomal‐transported circRNAs remain to be explored, and how m6A methylation participates in the regulatory mechanism still needs to be explored. It is worth thinking about how circPACRGL in tumor‐derived exosomes regulates miRNAs and TGF‐β1 to affect CRC; how miR‐17‐5p is assembled into exosomes and how circLONP2 participates in regulation have not been explored. Wnts derived from exosomal carcinoma‐associated fibroblasts can inhibit CRC chemosensitivity, which requires further investigation. Finally, this review summarizes that circRNAs can participate in the sponging of miRNAs, RNA transcription, protein translation, and direct binding to promote the invasion and metastasis of CRC and participate in the chemotherapy resistance of CRC, providing new drug therapeutic targets and biomarkers for the clinical treatment of CRC.

## ETHICAL APPROVAL STATEMENT

Not applicable.

## CONFLICT OF INTEREST

The authors declare that they have no conflict of interest.

## AUTHOR CONTRIBUTION

Shunhao Zhang, Jing Sun, and Minqi Gu were involved in the collection and collation of references. Shunhao Zhang wrote and edited the manuscript. Guihua Wang and Xudong Wang critically revised the manuscript for intellectual content. All authors read and approved the final manuscript.

## CLINICAL TRIAL REGISTRATION NUMBER

Not applicable.

## Data Availability

Not applicable.
